# Evidence of metabolic mechanisms playing a role in multiple insecticides resistance in *Anopheles stephensi* populations from Afghanistan

**DOI:** 10.1186/s12936-017-1744-9

**Published:** 2017-03-03

**Authors:** Noor Halim Zahid Safi, Abdul Ali Ahmadi, Sami Nahzat, Seyyed Payman Ziapour, Seyed Hassan Nikookar, Mahmoud Fazeli-Dinan, Ahmadali Enayati, Janet Hemingway

**Affiliations:** 1 National Malaria and Leishmania Control Programme, Ministry of Public Health, Kabul, Afghanistan; 2 Student Research Committee, Department of Medical Entomology and Vector Control, Health Sciences Research Center, School of Public Health, Mazandaran University of Medical Sciences, Sari, Iran; 3 Department of Parasitology, North Research Centre, Pasteur Institute of Iran, Amol, Iran; 4 Department of Medical Entomology and Vector Control, School of Public Health and Health Sciences Research Centre, Mazandaran University of Medical Sciences, Sari, Iran; 5 Liverpool School of Tropical Medicine, Liverpool, UK

**Keywords:** *Anopheles stephensi*, Insecticide resistance, Metabolic, Enzyme

## Abstract

**Background:**

Malaria is endemic in most parts of Afghanistan and insecticide-based vector control measures are central in controlling the disease. Insecticide resistance in the main malaria vector *Anopheles stephensi* from Afghanistan is increasing and attempts should be made to determine the underlying resistance mechanisms for its adequate management.

**Methods:**

The contents of cytochrome P450s, esterases, glutathione S-transferases (GSTs) and acetylcholine esterase (AChE) activities were measured in the Kunar and Nangarhar populations of *An. stephensi* from Afghanistan and the results were compared with those of the susceptible Beech strain using the World Health Organization approved biochemical assay methods for adult mosquitoes.

**Results:**

The cytochrome P450s enzyme ratios were 2.23- and 2.54-fold in the Kunar and Nangarhar populations compared with the susceptible Beech strain. The enzyme ratios for esterases with alpha-naphthyl acetate were 1.45 and 2.11 and with beta-naphthyl acetate were 1.62 and 1.85 in the Kunar and Nangarhar populations respectively compared with the susceptible Beech strain. Esterase ratios with para-nitrophenyl acetate (pNPA) were 1.61 and 1.75 in the Kunar and Nangarhar populations compared with the susceptible Beech strain. The GSTs enzyme ratios were 1.33 and 1.8 in the Kunar and Nangarhar populations compared with the susceptible Beech strain. The inhibition of AChE was 70.9 in the susceptible Beech strain, and 56.7 and 51.5 in the Kunar and Nangarhar populations. The differences between all values of the enzymes activities/contents and AChE inhibition rates in the Kunar and Nangarhar populations were statistically significant when compared with those of the susceptible Beech strain.

**Conclusions:**

Based on the results, the reported resistance to pyrethroid and organophosphate insecticides, and tolerance to bendiocarb in the Kunar and Nangarhar populations of *An. stephensi* from Afghanistan are likely to be caused by a range of metabolic mechanisms, including esterases, P450s and GSTs combined with target site insensitivity in AChE.

## Background

Malaria is endemic in Afghanistan. From a total population of 31 million, 8.5 million live in areas of high transmission and more than 15.4 million in areas of low transmission [1]. Major vectors of malaria in Afghanistan are *Anopheles stephensi, Anopheles culicifacies, Anopheles superpictus, Anopheles hyrcanus, Anopheles pulcherrimus,* and *Anopheles fluviatilis* [1], the first two being the most important in the country [2–6]. *An. stephensi* is widespread in different countries in the Middle East including Iran, Iraq, Bahrain, Saudi Arabia, Oman, India, Pakistan, Afghanistan, Bangladesh, South China and Myanmar (see [7]). Malaria in Afghanistan is predominantly due to *Plasmodium vivax* (95% of the cases) and *Plasmodium falciparum* (5%) in two distinct transmission seasons. The total number of confirmed cases in 2015 was 61362 [1].

As in many malaria endemic countries the main malaria control intervention was indoor residual spraying (IRS) with DDT (1950s to 1970), continued with organophosphate insecticides (OPs), such as malathion in later years, followed by insecticide-treated nets (ITNs) in the 1990s, and long-lasting insecticidal nets (LLINs) distribution from 2007 onwards [2, 3]. Deltamethrin-treated LLINs distribution to households in the main malaria-endemic provinces in Afghanistan is currently the main malaria control intervention [3]. During 2007–2014, the number of deltamethrin-treated LLIN distributed in Kunar and Nangarhar was 334,080 and 1,386,217, respectively [8]. Selection pressure from pesticides used in vector control and also in agriculture might have contributed to insecticide resistance in malaria vectors in Afghanistan especially in *An. stephensi.* This vector species from Nangarhar is resistant to DDT, bendiocarb, permethrin and deltamethrin, and to DDT, deltamethrin, permethrin and malathion in Kunar [8, 9]. Resistance to several insecticides including DDT, dieldrin, malathion and more recently pyrethroids have been reported in *An. stephensi* from the Middle East region [10–13] and in Afghanistan neighbouring countries including India [7]. The involvement of different enzymes and site insensitivity mechanisms in insecticide resistance in *An. stephensi* from Iran was confirmed [13–16]. *An. stephensi* from India had increased activities of esterases and GSTs associated with deltamethrin and permethrin resistance [17, 18]. Involvement of GSTs in insecticide resistance is evident in many insects including different mosquitoes [19]. General esterases are involved in OPs resistance in *An. stephensi* from Pakistan [20]. In recent years, World Health Organization (WHO) standard insecticide susceptibility bioassays have been performed on *An. stephensi* from Afghanistan showing resistance to organochlorines, carbamates and pyrethroid insecticides especially in the eastern provinces of Nangarhar and Kunar [4, 9, 21]. In addition, in 2014, target site insensitivity for pyrethroid insecticides known as knockdown resistance (KDR) was studied in *An. stephensi* from Kunar and Nangarhar. KDR is due to some single-nucleotide polymorphisms (SNP) causing voltage-gated sodium channels of the axons of the nerve cells to become insensitive to the knockdown effect exerted by pyrethroid insecticides. Although the wild type susceptible 1014L allele in the sodium channel gene was most prevalent followed by L1014S (*kdr east*, 21.4%) and L1014F (*kdr west*, 1.4%), no *kdr* homozygotes were detected. Only when the mutation data of *kdr*_e_ and *kdr*_w_ are combined, was there any significant association between *kdr* frequency and insecticide resistance. However, when they are separately considered, there was no significant association between *kdr* frequency and pyrethroid resistance. The finding that many of the bioassays survivors did not possess the *kdr* mutation suggests that other resistance mechanisms are present in these populations [21].

Accurate information on the underlying resistance mechanisms in *An. stephensi* is needed for proper management of insecticide resistance and a better management of malaria through vector control interventions. Therefore, based on the recommendations of the global plan for insecticide resistance management (GPIRM) [22], the need to develop an evidence-based malaria control plan and the suggestion that resistance mechanisms other than *kdr* are present in *An. stephensi* [21], other mechanisms operating in *An. stephensi,* the main malaria vector in Nangarhar and Kunar Provinces in eastern Afghanistan were investigated.

## Methods

### Study area

**Fig. 1:**
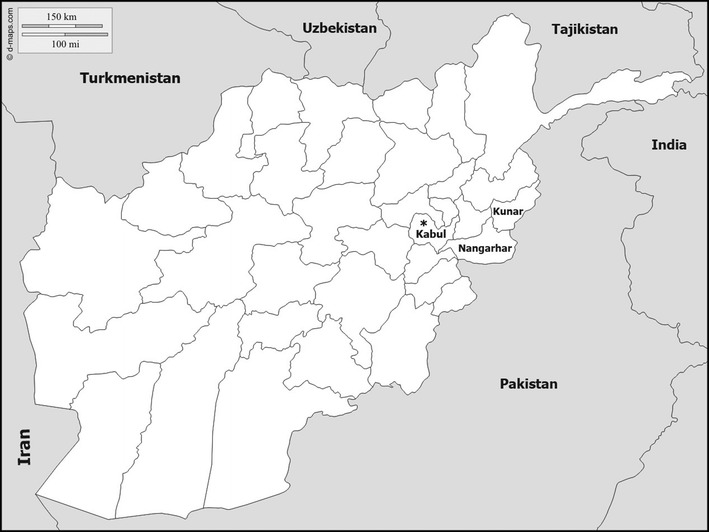
Map of Afghanistan and the location of the provinces of Nangarhar and Kunar in Northeastern region of the country where sampling for *Anopheles stephensi* took place in October 2015

The study area was the provinces of Nangarhar (34.1718°N, 70.6217°E) and Kunar (34.8466°N, 71.0973°E) in eastern Afghanistan (Fig. 1). The sampling places were the same exact districts, villages and coordinates where collections took place for the analysis of *kdr* in 2014 [21]. These sampling sites were 10 villages in Behsood, Jalalabad and Kama districts in Nangarhar; and Chawkay, Nurgal and Assadabad districts in Kunar (Table 1).

**Table 1:** Sampling places of *Anopheles stephensi* in Kunar and Nangarhar provinces, for biochemical assays of insecticide resistance in Afghanistan in 2015

Province	District	Village	No. of larvae
Nangarhar	Behsood	Banaghar, Samarkhel, Saracha	200
	Jalalabad	Bagrami	200
	Kama	Banajur, Sabirlalay, Sangarsary	200
Kunar	Chawkay	Babur	200
	Nurgal	Nurgal	200
	Assadabad	Asadabad	200

### Mosquito populations

Larvae of the Kunar and Nangarhar populations of *An. stephensi* were collected from the field (200 larvae from each district). They were reared to adults in the insectary located in Nangarhar National Malaria and Leishmaniasis Control Programme (NMLCP) field station. The adult mosquito specimens were identified to species using Glick’s identification keys [23]. Two to three days old adult mosquitoes were transported in cool boxes to NMLCP of the MoPH, Kabul. Upon arrival, they were immediately placed in a freezer (−80 °C) and remained there until transported on dry ice to the Pesticide Biochemistry Laboratory of Medical Entomology Department, School of Public Health, Mazandaran University of Medical Sciences, Sari, Iran for further analysis. The susceptible Beech strain of *An. stephensi* was provided by the Department of Medical Entomology, School of Public Health, Tehran University of Medical Sciences, Iran.

### Biochemical assays

The biochemical assays were performed according to the protocol of WHO/WHOPES (1998) [24–26]. The enzyme activity of glutathione S-transferases (GSTs) and esterases as well as the P450s contents and inhibition rates of acetylcholinesterase (AChE, using propoxur) were measured. All the assays were performed using appropriate buffer solutions which were prepared in advance and used within a maximum of 1–2 weeks after preparation. The remaining solutions and reagents were freshly prepared.

### Preparation of the mosquito homogenates

Individual deep-frozen adult mosquitoes were manually homogenized using a steel pestle in 300 µl cold 0.0625 M phosphate buffer pH 7.2 at 4 °C in flat bottom 96-well microtitre plate. The homogenates were centrifuged at 1109*g* (3000 rpm) at 4 °C for 20 min and the resulting supernatant was used as the enzyme source in all the enzyme reaction mixtures.

### Cytochrome P450s assay

In a fresh microtitre plate, the reaction mixture in each well consisted of 20 µl of the homogenate in duplicate, 80 µl of 0.0625 M potassium phosphate buffer pH 7.2, 200 µl of 3,3′,5,5′ tetramethylbenzidine (TMBZ) solution (0.01 g TMBZ dissolved in 5 ml methanol plus 15 ml of .25 M sodium acetate buffer pH 5.0) and 25 µl of 3% hydrogen peroxide. The absorbance was measured at 450 nm as an endpoint after incubating the plate at room temperature for 2 h. The enzyme contents were reported as equivalent units of cytochrome (EUC) P450s/mg protein corrected for the known haem content of cytochrome C and P450s using a standard curve of purified cytochrome C.

### General esterase assay

Alpha- and beta-naphthyl acetate were used to measure general esterase activity. Reaction mixtures contained 20 µl of the homogenate in duplicate (for each substrate) in adjacent microtitre plate wells (assigned alpha and beta) and 200 µl of alpha- or beta-naphthyl acetate solution (120 µl of 30 mM alpha- or beta-naphthyl acetate dissolved in 12 ml 0.02 M phosphate buffer pH 7.2) respectively. After incubating the mixtures at room temperature for 30 min, 50 µl of fast blue solution (0.023 g fast blue dissolved in 2.25 ml distilled water and 5.25 ml of 5% SDS .1 M sodium phosphate buffer pH 7) was added to each well. After another incubation period at room temperature for 5 min, the absorbance was measured at 570 nm as an endpoint. The resulting optical densities (OD) were converted to product concentration using standard curves of ODs for known concentrations of the products alpha- and beta-naphthol, respectively. The enzyme activities were reported as µM of product formed/min/mg protein.

### pNPA esterase assay

Ten microliter of the homogenate in duplicate was prepared in fresh 96-well microtitre plate to which 200 µl of pNPA working solution (100 mM pNPA in acetonitrile: 50 mM sodium phosphate buffer pH 7.4, 1:100) was added. Enzyme activity was measured kinetically at 405 nm for 2 min. The pNPA activity per individual was reported as µM of product formed/min/mg protein.

### GST assay

To a reaction mixture of 200 µl of reduced glutathione plus 1-chloro-2,4-dinitrobenzene (CDNB) solution (10 mM reduced glutathione dissolved in .1 M phosphate buffer pH 6.5 and 3 mM CDNB originally dissolved in methanol) 10 µl of the homogenate was added in duplicates. The absorbance was measured kinetically at 340 nm for 5 min. The enzyme activity was reported as mM of conjugate produced/min/mg protein using the extinction co-efficient of CDNB corrected for the path length of the solution in the microtitre plate well.

### Acetylcholinesterase (AChE) assay

The AChE in the 25 µl homogenates in duplicates was solubilized by adding 145 μl of Triton phosphate buffer (1% Triton X-100 in .1 M phosphate buffer pH 7.8) to each replicate. Ten µl of DTNB solution (0.01 M dithiobis-2-nitrobenzoic acid in .1 M phosphate buffer pH 7.0) and 25 µl of the substrate ASCHI (0.01 M acetylthiocholine iodide) were added to one replicate to initiate the reaction. The latter solution was substituted by 25 µl of the substrate ASCHI containing .2% of the inhibitor propoxur (.1 M) for the second test replicate. The kinetics of the enzyme reaction was monitored continuously at 405 nm for 5 min. The percentage of inhibition of AChE activity by propoxur in the test compared to the uninhibited wells was calculated. The assay conditions were preset so that individuals without an altered AChE-based resistance mechanism had >60% inhibition of the AChE activity.

### Protein assay

Protein content of each well was measured using Bradford method by adding 300 µl of Bio-Rad reagent (Bio-Rad, Italy), 1:4 diluted with ddH_2_O from stock to 10 µl of supernatant in duplicates. The absorbance was measured at 570 nm after the mixture was incubated for 5 min at room temperature. Absorbance was converted into protein concentration using a bovine serum albumin standard curve obtained with the same method and reagents. In all these biochemical assays, at least three blank replicates were prepared using all the reagents and solutions of each corresponding assays except adding distilled water instead of the enzyme source. The ODs of the test wells were corrected by subtracting with the average ODs of the blank replicates.

### Data acquisition

The reading of the activity/contents of the enzymes were done in a UV/visible microtitre plate reader (Bioteck, USA) run under KC junior software and the resulted data were directly extracted to the Microsoft Excel for further analysis. Mean values of activity or contents of each enzyme of all populations were compared employing ANOVA in conjunction with the Tukey’s statistical test using SPSS version 19 software. Enzyme ratios (ER) were calculated by dividing the mean activities or content of the field strains with those of the Beech susceptible strain.

### Data transformation and analyses

After performing a complete series of biochemical assays on *An. stephensi* mosquitoes from Kunar and Nangarhar from Afghanistan as well as the susceptible Beech strain, the data were transformed to the actual esterases (for alpha- and beta-naphthyl acetate, and pNPA), GSTs and AChE activities and cytochrome P450s contents. The activities of AChE of the replicates with and without propoxur are compared and the percentage inhibition is calculated. These values were compared with those of the Beech susceptible strain. One-way ANOVA/Tukey was used for the comparison of the mean values of the enzymes of different populations.

## Results

### Mosquito samples

Two hundred larvae were collected from each sampling area, reared to adults in insectary and morphologically identified *An. stephensi* specimens were used for biochemical assays.

### Cytochrome P450s contents

**Fig. 2:**
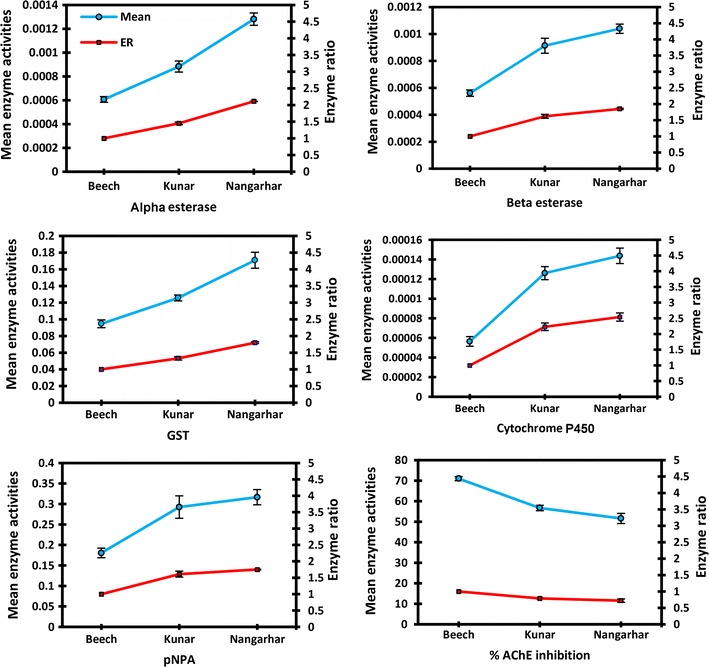
Mean enzyme activities and enzyme ratios (ER) measured in *Anopheles stephensi* populations from Nangarhar and Kunar provinces of Afghanistan compared with those of the susceptible Beach strain

The contents of cytochrome P450s in the Kunar and Nangarhar populations were 0.000126 and 0.000143 EUC cytochrome P450s/mg protein respectively, compared with 0.000056 in the susceptible Beech population (Table 2). The ratio of cytochrome P450s in the Kunar and Nangarhar populations were 2.25 and 2.55 when compared with that of the susceptible Beech strain (Table 3; Fig. 2). The differences of the contents of cytochrome P450s between the Nangarhar and Kunar populations were not statistically significant. However, the differences between the cytochrome P450s in those field populations were statistically significant at 5% level compared to the susceptible Beech strain (Table 4).

**Table 2:** Descriptive analysis of the results of the biochemical assays performed on *An. stephensi* populations from Afghanistan in 2016

		N	Mean	Std. deviation	Std. error	95% confidence interval for mean		Minimum	Maximum
						Lower bound	Upper bound		
Descriptives									
Alpha	1.0	90	0.000606935	0.0002243546	0.0000236491	0.000559945	0.000653926	0.0000613	0.0012599
	2.0	90	0.000882944	0.0004391187	0.0000462872	0.000790972	0.000974915	0.0001749	0.0026900
	3.0	90	0.001281330	0.0004898449	0.0000516342	0.001178734	0.001383926	0.0004359	0.0025958
	Total	270	0.000923736	0.0004865769	0.0000296121	0.000865435	0.000982037	0.0000613	0.0026900
Beta	1.0	90	0.000560930	0.0002256270	0.0000237832	0.000513674	0.000608187	0.0000217	0.0012485
	2.0	90	0.000912961	0.0005289397	0.0000557551	0.000802176	0.001023745	0.0000624	0.0027933
	3.0	90	0.001039151	0.0003281376	0.0000345887	0.000970423	0.001107878	0.0004121	0.0018416
	Total	270	0.000837681	0.0004314334	0.0000262562	0.000785987	0.000889374	0.0000217	0.0027933
GST	1.0	90	0.094644126	0.0445931787	0.0047005338	0.085304266	0.103983987	0.0057680	0.2404168
	2.0	90	0.125687826	0.0334270256	0.0035235179	0.118686671	0.132688981	0.0602100	0.1936805
	3.0	90	0.170861991	0.0906375391	0.0095540355	0.151878326	0.189845656	0.0100789	0.4758752
	Total	270	0.130397981	0.0687645517	0.0041848773	0.122158703	0.138637260	0.0057680	0.4758752
P450s	1.0	90	0.000056364	0.0000468496	0.0000049384	0.000046551	0.000066176	0.0000001	0.0002452
	2.0	90	0.000126040	0.0000635588	0.0000066997	0.000112728	0.000139352	0.0000297	0.0003423
	3.0	90	0.000143667	0.0000744365	0.0000078463	0.000128077	0.000159257	0.0000215	0.0004290
	Total	270	0.000108690	0.0000729523	0.0000044397	0.000099949	0.000117431	0.0000001	0.0004290
pNPA	1.0	90	0.180557226	0.1103588829	0.0116328477	0.157443005	0.203671446	0.0018530	0.5062369
	2.0	90	0.292412987	0.2596860355	0.0273733116	0.238022800	0.346803174	0.0043444	1.6858418
	3.0	90	0.316713874	0.1764954792	0.0186042570	0.279747611	0.353680136	0.0454042	0.7121251
	Total	270	0.263228029	0.2004405133	0.0121984212	0.239211509	0.287244548	0.0018530	1.6858418
aAChE	1.0	90	70.977705297	9.5683676804	1.0085945120	68.973649485	72.981761109	50.3058104	97.2588297
	2.0	90	56.690061678	12.527164551	1.3204792875	54.066297460	59.313825895	9.8746867	84.2042755
	3.0	90	51.579518613	23.323592773	2.4585221776	46.694487414	56.464549812	0.1056745	98.8008065
	Total	270	59.749095196	18.161457650	1.1052703914	57.573014562	61.925175830	0.1056745	98.8008065

*1* Beech susceptible strain, *2* Kunar population, *3* Nangarhar population, *alpha* alpha esterase, *beta* beta esterase, *GST* glutathione S-transferase, *P450s* cytochrome P450s, *pNPA* para nitrophenyl acetate

**Table 3:** Mean enzyme activities and enzyme ratios (ER) measured in *An. stephensi* populations from Afghanistan

Enzyme	Population	Mean	ER ± SE
Alpha esterase	Beech	0.000606935	1
	Kunar	0.000882944	1.45 ± 0.04
	Nangarhar	0.001281330	2.11 ± 0.005
Beta esterase	Beech	0.000560930	1
	Kunar	0.000912961	1.62 ± 0.06
	Nangarhar	0.001039151	1.85 ± 0.02
GST	Beech	0.094644126	1
	Kunar	0.125687826	1.33 ± 0.05
	Nangarhar	0.170861991	1.8 ± 0.025
Cytochrome P450s	Beech	0.000056364	1
	Kunar	0.000126040	2.23 ± 0.12
	Nangarhar	0.000143667	2.54 ± 0.13
pNPA	Beech	0.180557226	1
	Kunar	0.292412987	1.61 ± 0.09
	Nangarhar	0.316713874	1.75 ± 0.013
% AChE inhibition	Beech	70.977705297	1
	Kunar	56.690061678	0.79 ± 0.02
	Nangarhar	51.579518613	0.72 ± 0.05

% AChE inhibition is the percentage of acetylcholine esterase inhibition of the field populations compared with the Beech susceptible strain

**Table 4:** One way ANOVA performed on the biochemical assays results of *An. stephensi* populations from Afghanistan

Tukey HSD							
Dependent variable	(I) Group	(J) Group	Mean difference (I−J)	Std. error	Sig.	95% confidence interval	
						Lower bound	Upper bound
Multiple comparisons							
Alpha	1.0	2.0	−0.0002760081*	0.0000598212	0.000	−0.000416998	−0.000135018
		3.0	−0.0006743949*	0.0000598212	0.000	−0.000815385	−0.000533405
	2.0	1.0	0.0002760081*	0.0000598212	0.000	0.000135018	0.000416998
		3.0	−0.0003983867*	0.0000598212	0.000	−0.000539377	−0.000257396
	3.0	1.0	0.0006743949*	0.0000598212	0.000	0.000533405	0.000815385
		2.0	0.0003983867*	0.0000598212	0.000	0.000257396	0.000539377
Beta	1.0	2.0	−0.0003520302*	0.0000569834	0.000	−0.000486332	−0.000217728
		3.0	−0.0004782201*	0.0000569834	0.000	−0.000612522	−0.000343918
	2.0	1.0	0.0003520302*	0.0000569834	0.000	0.000217728	0.000486332
		3.0	−0.0001261898	0.0000569834	0.071	−0.000260492	0.000008112
	3.0	1.0	0.0004782201*	0.0000569834	0.000	0.000343918	0.000612522
		2.0	0.0001261898	0.0000569834	0.071	−0.000008112	0.000260492
GST	1.0	2.0	−0.0310436995*	0.0091575029	0.002	−0.052626648	−0.009460751
		3.0	−0.0762178646*	0.0091575029	0.000	−0.097800813	−0.054634916
	2.0	1.0	0.0310436995*	0.0091575029	0.002	0.009460751	0.052626648
		3.0	−0.0451741651*	0.0091575029	0.000	−0.066757114	−0.023591216
	3.0	1.0	0.0762178646*	0.0091575029	0.000	0.054634916	0.097800813
		2.0	0.0451741651*	0.0091575029	0.000	0.023591216	0.066757114
P450s	1.0	2.0	−0.0000696763*	0.0000093394	0.000	−0.000091688	−0.000047665
		3.0	−0.0000873032*	0.0000093394	0.000	−0.000109315	−0.000065291
	2.0	1.0	0.0000696763*	0.0000093394	0.000	0.000047665	0.000091688
		3.0	−0.0000176269	0.0000093394	0.144	−0.000039639	0.000004385
	3.0	1.0	0.0000873032*	0.0000093394	0.000	0.000065291	0.000109315
		2.0	0.0000176269	0.0000093394	0.144	−0.000004385	0.000039639
pNPA	1.0	2.0	−0.1118557613*	0.0286442515	0.000	−0.179366240	−0.044345282
		3.0	−0.1361566482*	0.0286442515	0.000	−0.203667127	−0.068646169
	2.0	1.0	0.1118557613*	0.0286442515	0.000	0.044345282	0.179366240
		3.0	−0.0243008869	0.0286442515	0.673	−0.091811366	0.043209592
	3.0	1.0	0.1361566482*	0.0286442515	0.000	0.068646169	0.203667127
		2.0	0.0243008869	0.0286442515	0.673	−0.043209592	0.091811366
aAChE	1.0	2.0	14.2876436193*	2.4228440225	0.000	8.577340447	19.997946792
		3.0	19.3981866838*	2.4228440225	0.000	13.687883512	25.108489856
	2.0	1.0	−14.287636193*	2.4228440225	0.000	−19.997946792	−8.577340447
		3.0	5.1105430644	2.4228440225	0.090	−0.599760108	10.820846237
	3.0	1.0	−19.398166838*	2.4228440225	0.000	−25.108489856	−13.687883512
		2.0	−5.1105430644	2.4228440225	0.090	−10.820846237	0.599760108

* The mean difference is significant at the 0.05 level

### General esterase activity

#### Alpha- and beta-esterase

Figure 2 gives the results of analysis of the two *An. stephensi* populations from Afghanistan compared to the susceptible Beech strain. The mean activity of alpha- and beta-naphthyl acetate were 0.00088 and 0.00091 µM/min/mg protein in the Kunar population, 0.00128 and 0.001 µM/min/mg protein in the Nangarhar population and 0.0006 and 0.00056 µM/min/mg protein in the susceptible Beech strain (Table 2). The enzyme ratio between the field populations and the susceptible population is calculated and illustrated in Fig. 2. These ratios for alpha-naphthyl acetate were 1.46 and 2.13, and for beta-naphthyl acetate were 1.62 and 1.78 in the Kunar and Nangarhar populations respectively. The ratios in the Nangarhar population are higher than those of the Kunar population for alpha- and beta-naphthyl acetate implying that the activities of the esterases in the Nangarhar population are higher than those in the Kunar population (Table 3). The differences between the activities of alpha-naphthyl acetate from the Kunar and Nangarhar populations were statistically significant with each other as well as with those of the susceptible Beech strain at 5% level. However, although the mean activity of beta-naphthyl acetate in the Nangarhar population is higher than that of the Kunar population, this difference was not statistically significant (Table 4).

### pNPA assay

The activities of pNPA esterase were .2924 and .3167 µM/min/mg protein in the Kunar and Nangarhar populations when compared with that of the susceptible Beech strain of .18055 µM/min/mg protein (Table 2; Fig. 2). The enzyme ratios between the Kunar and Nangarhar populations were 1.61 and 1.75 when compared with that of the susceptible Beech strain (Table 3). Although the pNPA activity was higher in the Nangarhar population compared with that of the Kunar population, this difference was not statistically significant (p = .673). However, the differences between the pNPA activities in the Kunar and Nangarhar populations were statistically significant when compared with that of the susceptible Beech strain (p < 0.001, Table 4).

### GSTs activity

The activity of GSTs was .12568, .17086 and 0.09464 mM/min/mg protein in the Kunar, Nangarhar and the susceptible Beech populations respectively (Table 2). The ratio between GSTs activities in those populations and the susceptible Beech strain was 1.32 and 1.8 respectively (Table 3). The activity of the GSTs in the Kunar and Nangarhar populations were significantly higher than that of the susceptible Beech strain (p = 0.002), and the difference of GSTs activity in the Nangarhar population was statistically significant from that of the Kunar population at 5% level (p > 0.001, Table 4).

### AChE inhibition

The AChE inhibition rate was 70.97% in the susceptible Beech strain, 56.91 in the Kunar population and 51.57 in the Nangarhar population (Table 2). The inhibition levels in the both field populations were lower than the threshold of 60% set for considering the AChE insensitive to propoxur. There were significant differences between the two populations in AChE inhibition when compared with that of the susceptible Beech strain (p > 0.001). However, the differences between the inhibition rates of AChE in the Kunar and Nangarhar populations were not statistically significant (p = 0.09, Table 4).

The differences between the activities/contents of all the enzymes measured in this study in the Kunar and Nangarhar populations were statistically significant compared with those of the susceptible Beech strain. However, although all the enzymes activities/contents measured in this study were higher in the Nangarhar population of *An. stephensi* than the Kunar population, only the differences between alpha-esterases and GSTs were statistically significant in those two populations (Table 4). The results lead to the conclusion that the strength of resistance in *An. stephensi* in Nangarhar should be slightly higher to multiple insecticides than the Kunar population.

## Discussion

*Anopheles stephensi* from Nangarhar and Kunar Provinces showed resistance to pyrethroids including deltamethrin and permethrin, malathion and slightly to bendiocarb [21]. In an attempt to address the possible underlying resistance mechanisms, the frequency of *kdr* allele in *An. stephensi* from Nangarhar and Kunar in Afghanistan was previously determined. The pattern of L1014S and L1014F mutations was similar to that observed in India with L1014S being more frequent than L1014F [7]. As *kdr* is recessive and no homozygote *kdr* individuals were observed, the researchers suggested that other resistance mechanisms are driving the pyrethroid resistance in the field populations [21]. That is exactly what has been undertaken in the present study by measuring the activities, contents or inhibition rates of the enzymes which could be responsible for the insecticide resistance in *An. stephensi* from Afghanistan.

The differences between activities of all enzyme groups including esterases alpha-, beta- and pNPA substrates, GSTs and cytochrome P450s in the Kunar and Nangarhar populations are higher than those of the susceptible Beech strain, indicating that esterases, GSTs and cytochrome P450s could all be involved in insecticide resistance in those field populations. The inhibition levels of AChE and the frequency of insensitive AChE individuals in the field populations are significantly higher than that of the susceptible Beech strain. Insensitive AChE would impact on the resistance to malathion and tolerance to bendiocarb in the field populations. The involvement of these enzyme groups in insecticide resistance is quite common in different insects especially mosquitoes [13–15, 19, 27–29]. Involvement of esterases and cytochrome P450s in pyrethroid resistance was reported in *An. stephensi* from Dubai and India [13, 17]. Esterases can also confer resistance to OPs and cross resistance to pyrethroids [13, 20, 30, 31].

Although bendiocarb may still be, at least partially, effective against *An. stephensi* in the area, close monitoring of the susceptibility by bioassay as well as biochemical assays are recommended as increasing the frequency of the insensitive AChE could increase bendiocarb resistance levels. Insecticide resistance management strategies are also recommended to postpone or otherwise dilute the resistance to carbamates in Kunar and Nangarhar Provinces [26].

The frequency of AChE insentivity in the Kunar population is slightly less than in the Nangarhar population. Based on this criterion, only the Nangarhar population may show resistance to OPs and carbamates. A similar pattern of AChE insensitivity was seen in *Anopheles albimanus* in Mexico [26], in Turkish populations of the *Anopheles maculipennis* [32], and *An. stephensi* from Iran [16]. This higher level of resistance in *An. stephensi* in the Nangarhar compared to the Kunar population, could be a result of different pesticides in use in agriculture and more importantly higher number of deltamethrin-treated LLIN distributed in recent years in Nangarhar compared with that in Kunar [8].

## Conclusions

Different enzyme groups are involved in the resistance to insecticides in *An. stephensi* from Nangarhar and Kunar Provinces in Afghanistan. This coupled with the results of an earlier study confirming the involvement of KDR mechanism at least in part in the pyrethroid resistance in this vector, reveals that insecticide resistance due to multiple mechanisms is increasing in the main malaria vector *An. stephensi* in Afghanistan. Therefore, close monitoring and evaluation of the impact of insecticide resistance on the vector control measures is needed.

## Abbreviations

AChE: acetylcholinesterase
ASCHI: acetylthiocholine iodide
CDNB: 1-chloro-2,4-dinitrobenzene
DDT: dichlorodiphenyltrichloroethane
DTNB: dithiobis-2-nitrobenzoic acid
EUC: equivalent units of cytochrome
GPIRM: global plan for insecticide resistance management
GST: glutathione S-transferases
ITN: insecticide-treated net
KDR: knockdown resistance
*kdr*_*e*_: knockdown resistance east
*kdr*_*w*_: knockdown resistance west
LLIN: long-lasting insecticidal nets
MoPH: Ministry of Public Health
NLMCP: National Malaria and Leishmaniasis Control Programme
OD: optical density
OPs: organophosphates
P450s: cytochrome P450s
pNPA: para-nitrophenyl acetate
SDS: sodium dodecyl sulfate
TMBZ: 3,3′,5,5′ tetramethylbenzidine
WHO: World Health Organization
WHOPES: World Health Organization Pesticide Evaluation Scheme
